# Commentary: Mismatch Repair Deficiency and Microsatellite Instability in Triple-Negative Breast Cancer: A Retrospective Study of 440 Patients

**DOI:** 10.3389/fonc.2021.735476

**Published:** 2021-09-29

**Authors:** Konstantinos Venetis, Nicola Fusco, Elham Sajjadi

**Affiliations:** ^1^ Division of Pathology, IEO, European Institute of Oncology IRCCS, Milan, Italy; ^2^ Department of Oncology and Hemato-Oncology, University of Milan, Milan, Italy

**Keywords:** triple-negative breast cancer (TNBC), mismatch repair (MMR), microsatellite instability (MSI), biomarkers, testing methods

## Introduction

The mismatch repair (MMR) system maintains the genomic stability through the correction of base mispairing generated during DNA replication (1). Its deficiency has a relevant role in the tumorigenesis and tumor progression of a subset of breast cancers (2).

In an interesting study, Ren and collaborators (3) focus the attention on triple-negative breast cancers (TNBC). Using MMR immunohistochemistry (IHC) and microsatellite instability (MSI) PCR on a retrospective cohort of 440 patients, the Authors found only 1 (0.2%) MMR-deficient (dMMR) case, showing loss of MSH2 alone and low-frequency MSI (MSI-L). No MSI-high (MSI-H) tumors were observed, although overall 14 (7.2%) samples were MSI-L. The Authors confirm the low incidence of dMMR/MSI-H (4) and the high rate of discrepancy between MMR IHC/MSI PCR in TNBC (5). Finally, their analyses revealed no significant associations between MSI-L and other clinicopathological and prognostic features.

The topic is of great importance considering the growing interest on the implementation of consistent MMR testing for prognostication, immune checkpoints inhibitors (ICI) prediction, and identification of therapy resistance/susceptibility in both adjuvant and neoadjuvant settings (6–8). To date, in the neoadjuvant setting, several clinical trials have examined the efficacy of programmed death-1/programmed death-ligand 1 (PD-1/PD-L1) blockade in early high-risk TNBC (9–11). The results of the KEYNOTE-522 study have recently led to the approval of pembrolizumab in combination with chemotherapy for patients with locally recurrent unresectable or metastatic TNBC whose tumors express PD-L1 with combined positive score (CPS) ≥10 (10). Despite these remarkable achievements, additional biomarkers would be helpful in this setting. Therefore, this elegant work by Ron et al. is an excellent opportunity to reflect on the possibilities and challenges of MMR analysis for patients with TNBC.

## Frequency and Spectrum of Mismatch Repair Alterations in TNBC

**Figure 1:**
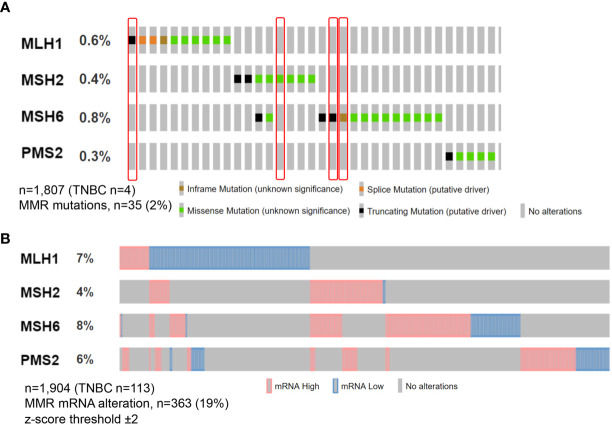
Oncoprint visualization of molecular alterations in the MMR genes in triple-negative breast cancer. Alterations across all breast cancer subtypes are color-coded on the basis of the legends on the bottom. Each column represents a sample, each row an MMR gene. Somatic MMR mutations **(A)** are seen in 35 (2%) of queried patients from MSK study with the majority showing missense mutation, while MMR mRNA alterations **(B)** in n = 363 (19%) from METABRIC study. Tumors included in this analysis have been retrieved from cbioportal.org.

Types of MMR alterations described in TNBC, include gene mutations, hypermethylation, RNA downregulation, and alterations in the expression patterns of the protein complexes (5, 12–16). The actual frequency of dMMR in TNBC, however, is controversial, since MMR mutations are reported in ∼2% of cases, while an impaired protein expression seems to be more frequent (15, 17, 18), probably due to post-transcriptional modifications (**Figure 1**). Interestingly, dMMR TNBC often present a single protein loss (19), as also noted by Ren et al.

## The Rationale for MMR Clinical Testing in TNBC

The previously reported significant prognostic role of dMMR in TNBC (6, 15) has not been confirmed by Ren et al. because they found only 1 IHC dMMR and no MSI-H cases. In this respect, a study by our group focusing on MMR patterns of expression showed a better prognosis for TNBC tumors with MMR proteins perturbations (5). Regarding the predictive role, although data on MMR alterations in TNBC are still being generated (20), the existing evidence is limited and therefore, further studies to establish its clinical value are expected. Hence, only a few TNBC were included in the basket trials that led to the ICI MMR-based histology-agnostic approval (21). Furthermore, the notion that relates the sensitivity to ICI to the adaptative immune response against neo-antigens, generated by super-mutator cancer cells, is another facet that needs further clarification in TNBC (22). Indeed, the tumor mutation burden observed in dMMR TNBC is overall lower than in other types of dMMR cancers, albeit significantly higher than in hormone receptor (HR)-positive breast cancers (2, 23). The interaction between MMR and other immune-related biomarkers in TNBC could be explored in the near future to improve a tailored MMR testing. Lately, it has been shown that dMMR TNBC preferentially show high stromal T-cell predominant tumor-infiltrating lymphocytes (TILs) and higher expression of PD-L1 and CD8 than those with an MMR proficient status (4, 24). In another study, patients with TILs-high TNBC revealed an inverse correlation between MLH1 and PD-L1 expression in stromal immune cells (25). As pointed by Ren et al., large multicentric cohorts are needed improve our understanding of the relationship between MMR and the other actionable biomarkers in TNBC.

## Currently Available Testing Methods and Guidelines

What we know so far is that MMR data in TNBC may vary according to the employed testing method, such as IHC for the four MMR proteins, MSI PCR, and next-generation sequencing (NGS) (26). Among these, IHC is usually employed as a first-line testing method due to its reliability, cost-effectiveness, and large availability (8, 27). Lately, we proposed phosphatase and tensin homolog (PTEN) as a complementary biomarker in breast cancer, as its wild-type expression by IHC had a 100% positive predictive value for MMR proficiency in several subtypes, including TNBC (16). MSI analysis using mononucleotide markers, also employed by Ren et al., is a highly sensitive method, albeit not specific for breast cancer (28–31). Given that NGS-based panels can screen a larger number of microsatellite loci compared to RT-PCR and allow for the simultaneous identification of other actionable genetic alterations, this technology is currently gaining momentum in cancers with lower MSI-H/dMMR frequency, such as TNBC (32–35). Regrettably, all these methods are generally molded on those approved for the archetypal Lynch syndrome tumors, where MSI occurs way more frequently than in TNBC (colorectal cancer predominantly) (36). To ensure optimal specificity and sensitivity in breast cancer, these diagnostic strategies might need to be re-developed or at least re-validated.

## Conclusion

The diagnosis and treatment of TNBC have remarkably progressed during the recent decades, yet many patients develop resistance to pharmacotherapy and die of this disease. The pathological identification of dMMR TNBC, albeit promising, has proven to be tremendously difficult due to the constraints of the existing methods and the scarcity of research. The study by Ren et al. represents another step forward in the discussion on the clinical utility of MMR testing in breast cancer. Further translational research studies and clinical trials encompassing tumor-specific guidelines for analytical and preanalytical phases are warranted to improve the characterization of the MMR status in TNBC.

## Author Contributions

All the authors equally participated in the writing and reviewing of the paper. All authors contributed to the article and approved the submitted version.

## Conflict of Interest

NF has received honoraria for consulting, advisory role, and/or speaker bureau from Merck Sharp & Dohme (MSD), Boehringer Ingelheim, and Novartis.

The remaining authors declare that the research was conducted in the absence of any commercial or financial relationships that could be construed as a potential conflict of interest.

## Publisher’s Note

All claims expressed in this article are solely those of the authors and do not necessarily represent those of their affiliated organizations, or those of the publisher, the editors and the reviewers. Any product that may be evaluated in this article, or claim that may be made by its manufacturer, is not guaranteed or endorsed by the publisher.
